# Pertussis outbreak investigation in Likimsa-Bokore *kebele*, Meda Walebu district, Bale zone, Oromia region, Ethiopia, 2019: a descriptive cross-sectional study

**DOI:** 10.11604/pamj.2024.48.37.20269

**Published:** 2024-05-31

**Authors:** Mohammed Hasen Badeso, Falaho Sani Kalil, Henok Asefa Ferede, Naod Berhanu Bogale

**Affiliations:** 1 Field Epidemiology Training Program, Faculty of Public Health, Jimma University, Jimma, Ethiopia,; 2 Department of Epidemiology, Faculty of Public Health, Jimma University, Jimma, Ethiopia

**Keywords:** pertussis, outbreak, Meda-Walebu, Bale, Southeast Ethiopia

## Abstract

**Introduction:**

pertussis is a major cause of childhood morbidity and mortality. Globally, an estimated 45 millions cases and 400,000 deaths occur every year. Meda Walebu surveillance office reported a pertussis outbreak among the residents of the Liqimsa-Bokore kebele communities. We investigated to describe the magnitude of the pertussis outbreak in Likimsa-Bokore kebele of Meda-Walebu district, Bale Zone, Southeast Ethiopia.

**Methods:**

we conducted a descriptive cross-sectional study in April 2019. We identified pertussis cases recorded on the line list. Suspected cases of pertussis were defined as any resident of Likimsa-Bokore kebele with cough illness and any of the following: paroxysms of coughing, inspiratory whooping, post-tussive vomiting, or apnea. The pentavalent vaccine coverage data were extracted from the Bale zone health management information system department database. Microsoft Excel pivot table and SPSS version 23 software cleaned and analyzed the data.

**Results:**

in three months period, a total of 439 suspected cases of pertussis were reported from Likimsa-Bokore kebele of the Meda-Walebu district. Half of the cases 220(50.1%) have occurred in females and the majority of cases 256 (58.3%) occurred in under five years children. The median age of cases was 4 years ranging from 2 months to 30 years (interquartile range= 4 years). The overall Attack Rate (AR) was 55 per 1000 population with a case fatality rate of 0.7% (3 deaths/439). Children less than five years were the most affected age group with an AR of 198 per 1000 population. The administrative pentavalent vaccine coverage of the district was above 100% during the year 2015-2018.

**Conclusion:**

the overall attack rate of pertussis outbreak was high. Children under five years were the most affected age group irrespective of high administrative coverage of the pentavalent vaccine. Strengthening routine immunization management and intensified surveillance system is required for early detection, investigation, and response activities.

## Introduction

Pertussis (whooping cough) is a contagious, respiratory disease caused by the bacterium *Bordetella pertussis*. The disease is endemic worldwide and cyclical increases every 2-5 years. The pertussis patient is typically characterized by symptoms of a prolonged paroxysmal cough that is often followed by an inspiratory whoop. The illness presentation can differ with age and history of prior exposure or vaccination. Young infants may present to the health facilities with apnea and no extra illness symptoms. Adults and adolescents with some immunity might exhibit solely gentle symptoms or have typically prolonged attack cough. In all persons, cough can continue for months. Pertussis rarely causes severe complications among healthy and vaccinated persons. However, infants are at the greatest risk for pertussis-related complications and mortality [1,2].

Globally, pertussis is a major cause of childhood morbidity and mortality. An estimated 45 million cases and 400000 deaths occur every year. Case-fatality rates in developing countries can reach 15% [3]. In 2015, 5.9 million under-5 deaths were reported, and of this pertussis contributed 0.9% [4]. In 2013, according to WHO estimates, pertussis was led to around 63000 deaths in children aged <5 years [5]. According to Global Health Statistics, 136,125 cases of pertussis was reported [6]. Additionally, in the final pertussis surveillance report of 2018 by the Center for Diseases Control and prevention (CDC), the total incidence of pertussis was 4.1 per 100,000 populations [7]. In Ethiopia, the maternal and newborn disparities country profile showed that pertussis contributes to 0.2% cause of neonatal death [8]. The study conducted in southwest Ethiopia indicated that the pertussis attack rate was 1.3 per 1000 population and the case fatality rate was 3.7 per 100 cases [9].

Vaccination remains the most effective way to prevent pertussis infection [10]. Immunization is one of the eleven Global Health Security Agenda (GHSA) action packages planned to support countries in developing sustainable immunization program capacity to prevent, detect, and respond to emerging disease threats including pertussis [11]. So high coverage with an effective routine vaccine is the backbone of pertussis prevention [3]. However, neither natural infection nor vaccination converses lifetime immunity against pertussis infection. As a result of waning immunity over time, adolescents and adults are susceptible to infection with *Bordetella pertussis*. The severity of pertussis is strongly linked to the time since previous vaccination or illness due to *Bordetella pertussis* [10,12]. According to the 2017 Global Routine Vaccine Coverage, global pentavalent vaccine coverage was 85%, accordingly in Africa and Ethiopia was 72% and 73% respectively [13]. Despite the widespread availability of pertussis vaccines and high vaccination coverage rates, pertussis continues to be a leading cause of death among children [2].

In the past, pertussis was primarily a disease affecting children of less than 6 years old. However, recently there has been a change in the epidemiology of pertussis such that, the disease also affects adolescents and adults. So, pertussis continues to be a global concern, even in countries with relatively strong economies and high rates of childhood immunization [12]. Surveillance statistics support the re-emergence of pertussis in developed countries. However, pertussis surveillance data is largely missing for low-income countries. Therefore, the epidemiology of pertussis in low-income countries is limited [14]. Despite the high immunization coverage of the administrative report, the outbreak was reported in Meda-Welabu district, Bale Zone. The pertussis outbreak occurred in the Meda-Walebu district, Likimsa-Bokore *kebele* communities from September 20 to December 15, 2018. Therefore, the focus of this study was to describe the magnitude of the pertussis outbreak in the Likimsa-Bokore *kebele* of the Meda-Walebu district, Bale Zone, Southeast Ethiopia.

## Methods

### Study area

Meda-Walebu district is found in the Bale zone which is 195 km from Robe Town, the capital of Bale zone, and 625 km from Addis Ababa located in southeast Ethiopia. According to the 2007 census and housing projection, the total population of the district in 2018 is estimated to be 134,371. Administratively, the Meda-Walebu district is divided into 20 *kebele*s and one administration town. The district shares the boundaries with Ethiopia Somali region and Guradhamole district on the East, the Guji zone in the south and west, Delomena district in the North. Regarding the health facilities, there are one district hospital, 6 health centers, and 21 health posts in the district. All these health facilities have been giving vaccination services to the communities. Likimsa-Bokore is one of the *kebele* of the Meda-Walebu district. This *kebele* has one health post and health center.

### Study design and period

We conducted a descriptive cross-sectional study in April 2019.

### Source of data and procedures

We identified pertussis case data from the pertussis outbreak line list in the database of the Public Health Emergency Management (PHEM) department, which is recorded during the outbreak. The pertussis cases were line-listed during an outbreak in the district and then, the line lists were reported to the Bale zone PHEM focal person on daily bases by the Meda-Walebu district health office PHEM unit. Accordingly, relevant variables such as date of symptoms onset, age, sex, clinical symptoms, and outcomes were extracted from the line list using a prepared data extraction format. Data related to pentavalent vaccination coverage were extracted from the Health Management Information System (HIMS) database of the Bale zone.

### Population

All populations in the Meda-Walebu district, Likimsa-Bokore *kebele* were our study population, and study subjects were all pertussis cases reported during the outbreak from September 20, 2018, to December 15, 2018.

### Case definition

**Suspected pertussis case:** any resident of Likimsa-Bokore *kebele* with cough illness and any of the following: paroxysms of coughing, inspiratory whooping, post-tussive vomiting, or apnea [1].

**Possible case:** a person who meets the suspected case definition but does not meet the confirmed classification, as defined above, should be considered a possible case. This includes suspected cases who did not have laboratory testing done and those who tested negative [1].

### Data analysis procedure

Data were checked for completeness and consistency and then, cleaned and analyzed using Microsoft Excel pivot table and SPSS version 23 software. Frequency, proportion, attack rate, and case fatality rate were calculated. The normal distribution of the continuous variable (age) was checked statistically using the Kolmogorov-Smirnov test. Accordingly, the age of the study participant was described in terms of median and interquartile range. The finding is summarized using a figure, table, and graph.

### Ethical consideration

We use secondary data to conduct this study. Accordingly, Jimma University wrote letters of support to conduct the study. From the Bale zonal health department, we received a letter granting us permission to utilize the data solely for our study.

## Results

**Figure 1:**
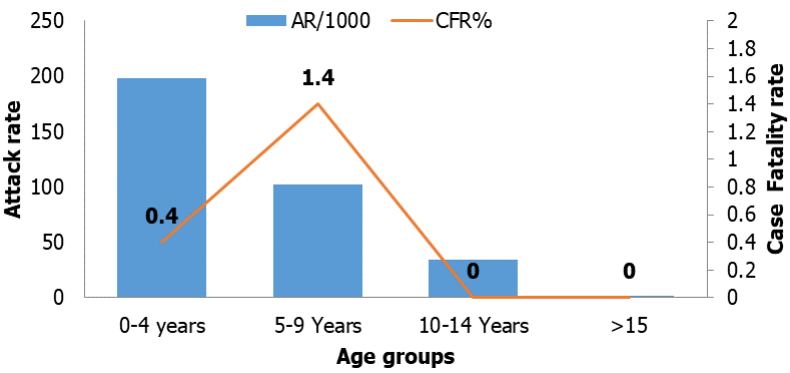
pertussis attack rate and case fatality rate by age group in Likimsa-Bokore *kebele*, Meda Walebu district, Bale zone, Ethiopia, April 2019

From September 20, 2018, to December 15, 2018, a total of 439 suspected cases of pertussis were reported from the Likimsa-Bokore *kebele* of the Meda-Walebu district. Half of the cases 220 (50.1%) occurred in females and the majority of cases 256 (58.3%) occurred in under five years children. The median age of cases was 4 years ranging from 2 months to 30 years and the interquartile range of 4 years. The overall Attack Rate (AR) was 55 per 1000 population with a case fatality rate of 0.7% (3 deaths/439). Children less than five years were the most affected age group with an AR of 198 per 1000 population. The attack rate in males and females was 57/1000 population and 54/1000 population respectively. Out of 3 deaths from pertussis, two occurred among females (Table 1). The attack rate among the age group 0-4 years was 198 per 1000 population of the *kebele* followed by 102 per 1000 population among the age group 5-9 years. The high case fatality rate occurred among the age group 5-9 years which is 1.4% (2 death/140 cases) case fatality rate (Figure 1).

**Table 1:** number of pertussis cases by age and sex in Likimsa-Bokore *kebele*, Meda Walebu district, Bale zone, Ethiopia, April 2019

Age groups	Male (Number, %)	Female (Number, %)	Total (Number, %)
Less than 4 years	121(27.6%)	135(30.8%)	256(58.3%)
5-9 years	76(17.3%)	64(14.6%)	140(31.9%)
10-14 years	19(4.3%)	18(4.1%)	37(8.4%)
Greater than 15 years	3(0.7%)	3(0.7%)	6(1.6%)
Total	219(49.9%)	220(50.1%)	439(100%)

**Figure 2:**
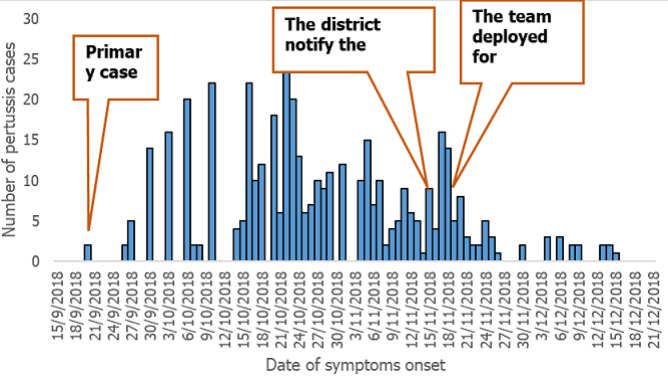
epi curve showing the number of pertussis cases and date of symptoms onset in Likimsa-Bokore *kebele*, Meda Walebu district, Bale zone, Ethiopia, April 2019

Meda-Walebu district surveillance office notified the pertussis cases Bale zone health department on November 16, 2018. The team from the Bale zone health office of the public health emergency management department was deployed to the field to assess the situation on November 18, 2018. The primary case of pertussis was the 6 years old female who is a resident of Likimsa-Bokore *kebele* of Meda-Walebu district, seen at the health facility on October 29, 2018, with the date of symptom onset on September 20, 2019. The cases started to build up on September 26, 2018, and fluctuated until November 17, 2018, then started to decrease until zero reports. The last case date of symptom onset was 15/12/2018 (Figure 2).

**Figure 3:**
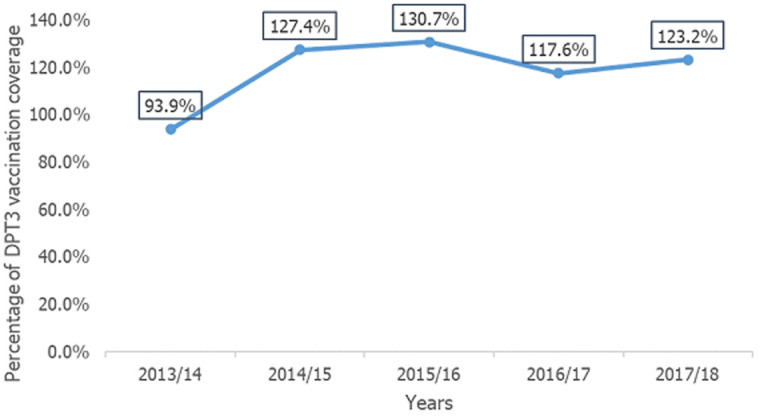
trend of DPT3 vaccination coverage of Meda Walabu district, Bale zone, Oromia region, Ethiopia, 2019

The result of the epidemic curve shows a propagative epidemic with multiple peaks. Concerning signs and symptoms of the cases, all cases (100%) have a paroxysmal cough, 98.2% have vomiting after the cough and 98.8% have chest pain. The administrative pentavalent vaccine coverage of the district was above 100% during the year 2015 to 2018 (Figure 3).

## Discussion

Pertussis is a vaccine-preventable disease. A reappearance both in infant and adult pertussis cases has been observed in many countries after the introduction of generalized vaccination. An antigenic difference between circulating bacteria and vaccinal strains, and inappropriate cold chain management, could be due to this resurgence [15]. Among the total cases reported during this outbreak, the proportion of females was slightly higher than that of males this was comparable with that of the pertussis outbreak that occurred in Canada [16] but reverse as the outbreak occurred in Aleppo [17]. Among the age groups, those 0-4 year age groups are more affected followed by the 5-9 year age group this is similar as compared to the study done in Northwest Ethiopia, South Wolo zone [9]. Additionally, consistent with the study conducted in Aleppo [17] but the study in Brunswick was contrary [18]. Our finding may be due to inappropriate vaccine management because pentavalent 3 vaccine coverage of the district was high according to the administrative data reviewed from the Bale zone health management information system.

This pertussis outbreak almost affects all age groups which is ranging from 2 months to 30 years. However, more than half of cases are occurred in under five age groups. The attack rate was higher as compared to the study conducted in Northwest Ethiopia, South Wolo [9], and Ghana [19]. Sex-specific attack rates both for males and females were higher as compared to the study conducted in Ghana [19]. The case fatality rate was lower as compared to the study conducted in Wolo this may be due to good case management during this outbreak [9]. The case fatality rate was high in the 5-9 years age groups this is supported by the body of science as the severity of pertussis depends on the time since previous vaccination [14]. So, this age group may take time since vaccination.

All cases identified during this outbreak had a paroxysmal cough. This study finding is greater as compared to the study conducted in Brunswick [18]. Most of the cases identified have vomiting after cough and chest pain. This finding is supported by the body of science that whooping cough is followed by vomiting [1]. Even though the vaccine coverage of the *kebele* was 123.2%, the outbreak has occurred, this may be due to inappropriate vaccine cold chain management which may result in vaccination failure. The district notified the outbreak to the next level which is the Bale zone health office after death was reported. This may be due to the weak surveillance system of the district which leads to the late detection and response to the pertussis outbreak that occurred in the district.

Our study is not free of limitations, of these, this study is limited by being a retrospective study as we were relying only on already collected secondary data. There was a possibility of some of the data might not correctly be recorded as the data was collected by the local health workers. Additionally, there was a limited variable in the reporting format. We were unable to determine the accurate immunization status of the case, in part due to incomplete records. As the cases recorded on the line list were not confirmed by the laboratory by collecting swabs, there might be a possibility of cases becoming truly pertussis cases.

## Conclusion

Pertussis attack rate is high in under five age groups and the case fatality rate was high in the female and 5-9 years age group. All reported cases have whooping cough and most of the cases have posttussive vomiting. There was a delay in the detection and notification of the outbreak to the next level by the district health office. Despite high pentavalent vaccine coverage, the outbreak occurred. Even though high coverage of vaccines, a pertussis outbreak occurred, so the district should strengthen the vaccination program and we recommend the researcher conduct a study on vaccine effectiveness in the district. The district should intensify the surveillance system for early detection, investigation, and response activities. We recommend further studies like case-control studies which determine the factors for the occurrence of a pertussis outbreak.

### What is known about this topic

- *Pertussis is a vaccine-preventable disease;*
- *Pertussis disease is endemic worldwide and cyclical increases every 2-5 years;*
- *Pertussis disease contributes to 0.2% cause of neonatal death in Ethiopia*.

### What this study adds

- *Pertussis outbreak occurred irrespective of high administrative pentavalent 3 vaccine coverage;*
- *Pertussis attack rate was high in children under five years of age;*
- *The case fatality rate of pertussis disease was high in females and children of 5-9 years*.

## References

[1] World Health Organization (2018). Pertussis: Vaccine-preventable diseases surveillance standards. World Health Organization.

[2] Drotman DP (2017). Emerging infectious diseases. Peer-Reviewed Journal Tracking and Analyzing Disease Trends. Center for Disease Control and prevention. Global health security.

[3] World Health Organization WHO Recommended Surveillance Standards. WHO and UNAIDS.

[4] Liu L, Oza S, Hogan D, Chu Y, Perin J, Zhu J (2016). Global, regional, and national causes of under-5 mortality in 2000-15: an updated systematic analysis with implications for the Sustainable Development Goals. Lancet.

[5] World Health Organization (2015). Pertussis vaccines: WHO position paper-August 2015. Wkly Epidemiol Rec.

[6] World Health Organization (2015). World Health Statistics 2015. World Health Organization.

[7] Center for Disease Control and prevention (2019). Provisional Pertussis Surveillance Report. Provisional 2018 Reports of Notifiable Diseases. CDC, National Center for Immunization and Respiratory Diseases. Division of Bacterial Diseases.

[8] Unicef Ethiopia (2016). Maternal and Newborn Disparities.

[9] Alamaw SD, Kassa AW, Gelaw YA (2017). Pertussis outbreak investigation of Mekdela district, South Wollo zone, Amhara region, North-West Ethiopia. BMC Res Notes.

[10] Maryland Department of Health and Mental Hygiene (2013). Local Health Department Guidelines for the Epidemiological Investigation and Control of Pertussis. Center for Immunization. Maryland Department of Health and Mental Hygiene.

[11] Centers for Disease Control and Prevention (2016). CDC's Strategic Framework for Global Immunization 2016-2020.

[12] Kilgore PE, Salim AM, Zervos MJ, Schmitt HJ (2016). Pertussis: Microbiology, Disease, Treatment, and Prevention. Clin Microbiol Rev.

[13] VanderEnde K, Gacic-Dobo M, Diallo MS, Conklin LM, Wallace AS (2018). Global Routine Vaccination Coverage-2017. MMWR Morb Mortal Wkly Rep.

[14] Muloiwa R, Kagina BM, Engel ME, Hussey GD (2015). The burden of pertussis in low-and middle-income countries since the inception of the Expanded Programme on Immunization (EPI) in 1974: a systematic review protocol. Syst Rev.

[15] World Health Organization Pertussis Vaccines. WHO Position Paper. Abstracts of references are provided in the position paper and GRADE tables. World Health Organization.

[16] Morton T, Birtwistle C, Fumerton R, Allison S (2018). Large pertussis outbreak in rural Canada: Lessons learned from Haida Gwaii. Can Fam Physician.

[17] Assistant coordination unit health department (2015). Pertussis Outbreak in Eastern Rural of Aleppo. AFP Surveillance; Early warning alert and response network.

[18] Office of the Chief Medical Officer of Health (2014). Pertussis outbreak investigation report. New Brunswick Department of Health.

[19] Field Epidemiology training program (2018). 67^th^ Annual Epidemic Intelligence Service Conference April 16-19 2018 U.S. Department of Health and Human Services. Centers for Disease Control and Prevention, Hilton Atlanta, 255 Courtland St NE, Atlanta, GA 30303.

